# Periodontal disease and risk of Alzheimer's disease: A two‐sample Mendelian randomization

**DOI:** 10.1002/brb3.3486

**Published:** 2024-04-22

**Authors:** Conglei Hu, Hui Li, Liping Huang, Rui Wang, Zeyu Wang, Rui Ma, Bei Chang, Shiting Li, Hongcai Li, Guangwen Li

**Affiliations:** ^1^ Graduate School Air Force Medical University Xi'an China; ^2^ Department of Epidemiology and Statistics School of Public Health, Southwest Medical University Luzhou China; ^3^ Department of Oral Implantology The Affiliated Stomatological Hospital, Southwest Medical University, Luzhou Key Laboratory of Oral & Maxillofacial Reconstruction and Regeneration Luzhou China; ^4^ Institute of Stomatology Southwest Medical University Luzhou China; ^5^ Department of Medical Education Tangdu Hospital, Air Force Medical University Xi'an China; ^6^ State Key Laboratory of Military Stomatology & National Clinical Research Center for Oral Diseases & Shaanxi Key Laboratory of Oral Diseases School of Stomatology, Air Force Medical University Xi'an China; ^7^ Department of Stomatology The PLA Rocket Force Characteristic Medical Center Beijing China; ^8^ Department of Stomatology Shuguang Hospital Affiliated to Shanghai University of Traditional Chinese Medicine Shanghai China

**Keywords:** Alzheimer's disease, Mendelian randomization, periodontal disease, SNPs

## Abstract

**Background:**

Evidence from observational studies and clinical trials suggests an association between periodontal disease and Alzheimer's disease (AD). However, the causal relationship between periodontal disease and AD remains to be determined.

**Methods:**

We obtained periodontal disease data from the FinnGen database and two sets of AD data from the IEU consortium and PGC databases. Subsequently, we conducted a two‐sample Mendelian randomization (MR) analysis to investigate the causal relationship between periodontal disease and AD.

**Results:**

The results of the random‐effects IVW analysis revealed no evidence of a genetic causal relationship between periodontal disease and AD, regardless of whether the AD data from the IEU consortium or the AD data from the PGC database were utilized. No heterogeneity, multiple effects of levels, or outliers were observed in this study.

**Conclusions:**

Our findings indicate that there is no causal relationship between periodontal disease and AD at the genetic level.

## Graphical abstract

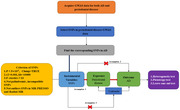

## 1 INTRODUCTION

Periodontal diseases encompass a spectrum of inflammatory conditions that compromise the supporting structures of the dentition, namely, the gingiva, alveolar bone, and periodontal ligament. Such afflictions carry the potential for tooth exfoliation and are implicated in eliciting systemic inflammatory responses. Chronic periodontitis prevalently targets the adult population, whereas aggressive periodontitis is an infrequent but documented occurrence among pediatric cohorts. The etiology of periodontal disease is intrinsically associated with dysbiosis within the symbiotic oral microbiome, chiefly manifested as dental plaque. This dysregulation precipitates an inflammatory cascade and disease formation as a consequence of the interplay with the host's immune defense mechanisms. Absent clinical intervention, the pathological cycle oscillates between phases of activity and quiescence until such time that the involved tooth is subject to extraction or resolution is achieved through the targeted disruption of the microbial biofilm, thus permitting the regression of inflammation. The intensity and progression of periodontal disease are subject to an intricate interdependence on both environmental and host‐derived risk determinants. These encompass factors of a mutable nature, such as smoking habits, as well as immutable variables like genetic predisposition (Kinane et al., 2017). According to global burden of disease estimates, severe periodontitis affects approximately 11% of the global adult population (Kassebaum et al., 2014). In 2018, the economic impact of periodontitis amounted to approximately $154 billion in the United States and €159 billion in Europe, imposing a substantial burden on both individuals and society (Botelho et al., 2022). supporting information.

Alzheimer's disease (AD) is a multifaceted neurodegenerative disorder characterized by the progressive accumulation of beta‐amyloid (Aβ) plaques and tau neurofibrillary tangles within the brain parenchyma, which are closely associated with widespread cerebral inflammatory processes (Li, 2023; Small et al., 1997). These pathological markers contribute to the gradual deterioration of cognitive function and the development of progressive memory impairments. Globally, dementia afflicts over 55 million individuals, with AD accounting for approximately 50–60% of all dementia cases (Blennow et al., 2006; GBD 2019 Dementia Forecasting Collaborators, 2022). Projections suggest that the number of individuals affected by Alzheimer's disease and other forms of dementia will reach approximately 152 million by 2050(GBD 2019 Dementia Forecasting Collaborators, 2022). In 2019, the estimated global cost of AD amounted to $2.8 trillion, with this figure expected to escalate to $16.9 trillion by 2050 (Nandi et al., 2022), imposing significant emotional and economic burdens on both families and society (Wimo et al., 2017).

Epidemiological investigations have established a significant relationship between Alzheimer's disease (AD) and the presence of periodontitis (Chen et al., 2017; Yu & Kuo, 2008). The gut‐brain axis has revealed an association between the gut microbiota and cognitive decline, suggesting a possible involvement of bacteria in AD pathogenesis (Lyte et al., 2006; Rhee et al., 2009). The oral cavity, hosting over 700 microbial species, represents the second largest reservoir of microorganisms after the gut (Chen et al., 2010). The oral‐brain axis provides direct and indirect evidence linking oral microbiota to immune mechanisms in the brain, especially with regard to periodontal pathogens (Narengaowa et al., 2021). While the precise mechanisms remain to be elucidated, postmortem brain tissue studies have indicated the presence of various bacteria, such as *Porphyromonas gingivalis* and *Treponema denticola*, which are integral components of the oral microbiome, in AD patients (Dominy et al., 2019; Poole et al., 2013; Riviere et al., 2002). Notably, animal models and research by Ishida et al. (2017) have demonstrated the ability of periodontal pathogens to access the brains of mice, potentially exacerbating AD's pathological characteristics and contributing to its progression (Poole et al., 2015).

As an emerging approach, Mendelian randomization (MR) has been employed to investigate potential causal links between exposure factors and outcomes. MR utilizes specific single nucleotide polymorphisms (SNPs) as instrumental variables (IVs) (Emdin et al., 2017). Due to the random distribution of alleles during gamete formation, this design is less susceptible to confounding or reverse causality. Leveraging the strengths of this study design, MR can effectively elucidate the causal relationship between exposure and outcome. With the continuous emergence of data from large‐scale genome‐wide association studies (GWAS), methods for attaining robust statistical power have become increasingly effective. Hence, this study employs a two‐sample Mendelian randomization approach to explore the potential causal relationship between periodontal disease and AD, aiming to provide a theoretical basis for the association between these two conditions.

## 2 METHOD

### 2.1 Study design

This MR study is based on a large‐scale GWAS pooled dataset. Informed consent has been obtained from all subjects in the original studies. Additional ethical approval was not required as we used summary‐level statistics. The primary analysis involved the utilization of periodontal disease data from the FinnGen consortium (*n* = 199,515) and Alzheimer's disease (AD) data from both the IEU consortium (*n* = 488,285) and the PGC database (*n* = 1,126,563).

Two independent validations were conducted, with periodontal disease from the FinnGen consortium as the exposure factor and AD from the IEU consortium as the outcome, and periodontal disease from the FinnGen consortium as the exposure factor and AD from the PGC database as the outcome. Sensitivity analyses included Cochran's *Q* test, leave‐one‐out analysis, funnel plot, and MR‐Egger intercept analysis. In the presence of heterogeneity or pleiotropy, outlier detection was performed using the Radial MR and MR‐pleiotropy residuals and outliers method (MR‐PRESSO). After removing outliers and estimating causal effects, multiple random‐effects IVW analysis was conducted to detect heterogeneity.

**FIGURE 1:**
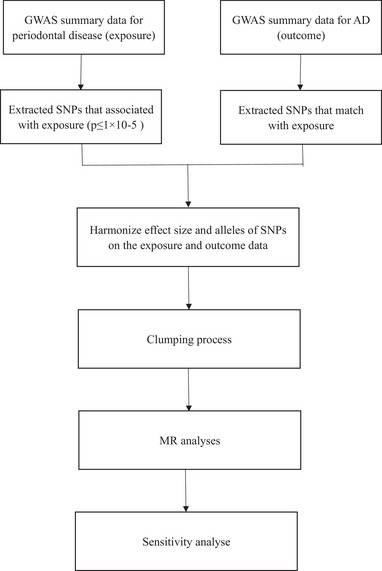
Study design and work flow. GWAS, Genome‐Wide Association Studies; AD, Alzheimer's disease; SNP, single nucleotide polymorphism; MR, Mendelian randomization.

A two‐sample Mendelian randomization (MR) study necessitates the satisfaction of three crucial assumptions. The first assumption postulates a close association between genetic instrumental variation and periodontal disease (as an exposure factor). Based on the second assumption, the genetic instrumental variation of periodontal disease should remain unaffected by any confounding factors owing to the random allocation of SNPs during gametogenesis. The third assumption asserts that the genetic instrumental variations in periodontal disease exert a significant influence on the risk of the outcome (AD) primarily through exposure to periodontal disease and not through other pathways. The aforementioned second and third assumptions are collectively referred to as the pleiotropic irrelevance hypothesis (Emdin et al., 2017). Study design and work flow can be found in Figure 1.

### 2.2 Genetic variants associated with periodontal disease

The main genetic instrument was derived from the periodontal disease GWAS dataset in the recent FinnGen database, which included 199,515 individuals of European ancestry. Given some limitations of this dataset, we only selected genome‐wide genetic variation (*p* < 1× 10^–5^) as a potential research tool. To obtain SNPs that are independent of each other, we trimmed these instruments within a window size of 10,000 kb to reduce the impact of *r*2 < 0.001 in linkage disequilibrium (LD). The *F*‐statistic was used to assess the strength of the correlation between instrument variables and exposure factors. Only when the *F*‐statistic is greater than 10 do we consider that weak instrumental variables do not cause bias (Pierce et al., 2011).

### 2.3 GWAS summary data for AD

We utilized pooled GWAS data for Alzheimer's disease (AD) obtained from the IEU consortium and the PGC database. The IEU consortium dataset comprised 954 AD cases and 487,331 controls, whereas the PGC database dataset encompassed a total of 1,126,563 samples (Wightman et al., 2021).

### 2.4 Statistical analysis

We extracted single nucleotide polymorphisms (SNPs) associated with exposure from the complete genome‐wide association study (GWAS) data of Alzheimer's disease (AD) and ensured allele alignment between exposure and outcome SNPs. We excluded SNPs with incompatible alleles or intermediate allele frequencies in palindromic sequences. This procedure involved four steps. First, SNPs were clustered to obtain independent genetic instrumental variables. Second, missing SNPs were replaced with proxy SNPs. Third, SNPs significantly associated with the outcome were excluded. Fourth, ambiguous and palindromic SNPs were discarded. Subsequently, Mendelian randomization (MR) analysis was conducted. Specifically, we employed the inverse variance weighting (IVW) method to estimate the primary MR effect, which was reported as an odds ratio (OR) with a 95% confidence interval (CI) (Burgess et al., 2013). Additionally, we employed the weighted median method and MR‐Egger regression to estimate the causal effects. These three methods are widely acknowledged and commonly employed for robust analysis of Mendelian randomization study results (Chen et al., 2021; Chen et al., 2022). If the weighted median method is utilized, at least 50% of the SNPs must meet the assumption of being valid instrumental variables (Bowden et al., 2016). The MR‐Egger regression adjustment allows detection of violations in the standard instrumental variable assumptions and provides an effect estimate that is not influenced by such violations (Ong & MacGregor, 2019).

Sensitivity analyses are essential for evaluating potential biases in Mendelian randomization studies. These analyses consider two factors: heterogeneity testing and pleiotropy testing. Heterogeneity was assessed using the Cochran's *Q* test in the IVW method, and the presence of pleiotropy was indicated by the intercept in MR‐Egger regression (an intercept with a *p*‐value less than.05 was considered indicative of pleiotropy) (Bowden et al., 2015; Burgess & Thompson, 2017). Additionally, we employed the MR‐PRESSO and Radial MR methods to eliminate SNPs with pleiotropic outliers (*p*‐values less than.05) (Bowden et al., 2018; Ong & MacGregor, 2019). In cases where potential outliers were identified, they were excluded, and the IVW estimation was repeated to evaluate the robustness of our findings. Moreover, we conducted a multiplicative random‐effects IVW analysis. We also performed a leave‐one‐out analysis to assess whether a single SNP was driving or biasing the MR estimate. As the name suggests, SNPs were systematically excluded, and the MR analysis was repeated to evaluate if the causal estimate was primarily influenced by a single SNP.

The MR analysis was performed using R software (version 4.3.1), TwoSample MR package (version 0.5.7), along with Storm Statistical Platform (www.medsta.cn/software).

## 3 RESULTS

### 3.1 Results of exposure factors for periodontal disease with the IEU Consortium AD dataset

In our study, we effectively extracted 17 genetic variants linked to periodontal disease by harmonizing the AD dataset from the IEU consortium. Our analysis yielded a mean *F*‐statistic value of 20.97, surpassing the conventional threshold of 10. This outcome signifies our successful mitigation of bias arising from weak instrumental variables.

**FIGURE 2:**
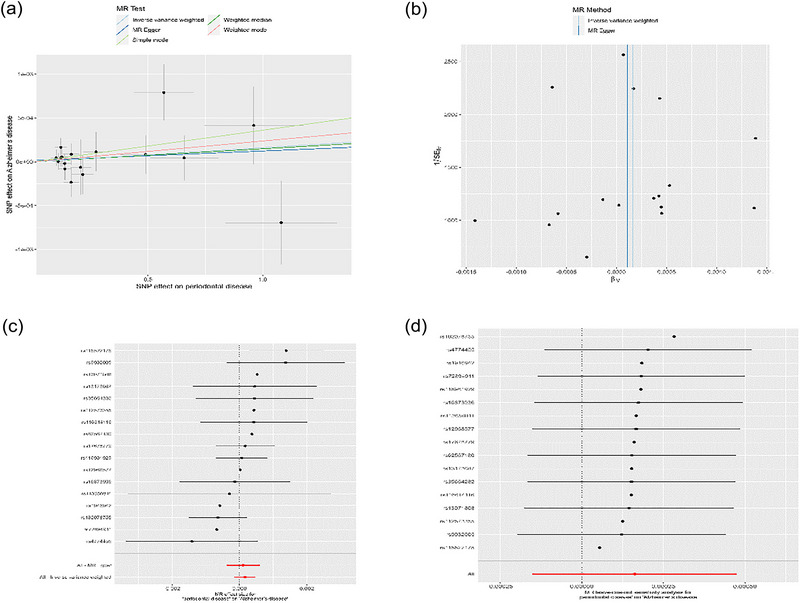
Mendelian randomization estimates from periodontal disease for AD (IEU Consortium). (a) Scatter plot showing the causality of periodontal disease on AD. (b) Funnel plots. (c) Forest plots of the IVW estimates. (d) Leave‐one‐out plots.

We employed IVW, MR‐Egger regression, and weighted median approaches to evaluate the causality between periodontal disease and AD. The IVW analysis indicated no causal relationship between periodontal disease and the risk of AD (OR = 1.00, 95% CI = 0.99‐1.00, *p* = .31). Both MR‐Egger regression and weighted median methods yielded consistent findings. To ensure the study's rigor and comprehensiveness, we conducted sensitivity analyses. Cochran's *Q* test provided no evidence of heterogeneity (*Q* value = 14.84, *p*‐value = .537). The horizontal pleiotropy test revealed no significant intercept (intercept = 1.51e‐5, *p* = .783), suggesting the absence of horizontal pleiotropy. Summary results of the horizontal pleiotropy test and the heterogeneity test can be found in Table 1. We performed outlier screening, but no outliers were identified, and thus the MR‐PRESSO method and Radial MR method were not employed. Leave‐one‐out analysis was conducted to verify each SNP's impact on the overall causal estimation. Figure 2 presents the leave‐one‐out plot and other results.

**TABLE 1:** Sensitivity analyses of periodontal disease IVs for AD (IEU Consortium).

Pleiotropy test			Heterogeneity test					
MR‐Egger			IVW			MR‐Egger		
Intercept	SE	*p*	*Q*	*Q*_df	*Q*_pval	*Q*	*Q*_df	*Q*_pval
1.51 × 10^−5^	5.4 × 10^−5^	.783	14.835	16	0.537	14.756	15	0.469

MR, Mendelian randomization; IVW, inverse variance weighted; SE, standard error.

### 3.2 Results of exposure factors for periodontal disease and the PGC database for the AD dataset

In our study, we successfully identified 12 genetic variants associated with periodontal disease by harmonizing the AD dataset obtained from the PGC database. Our analysis revealed that the average *F*‐statistic value was 21.08, surpassing the conventional threshold of 10. This signifies our successful resolution of the issue of weak instrumental variable bias.

**FIGURE 3:**
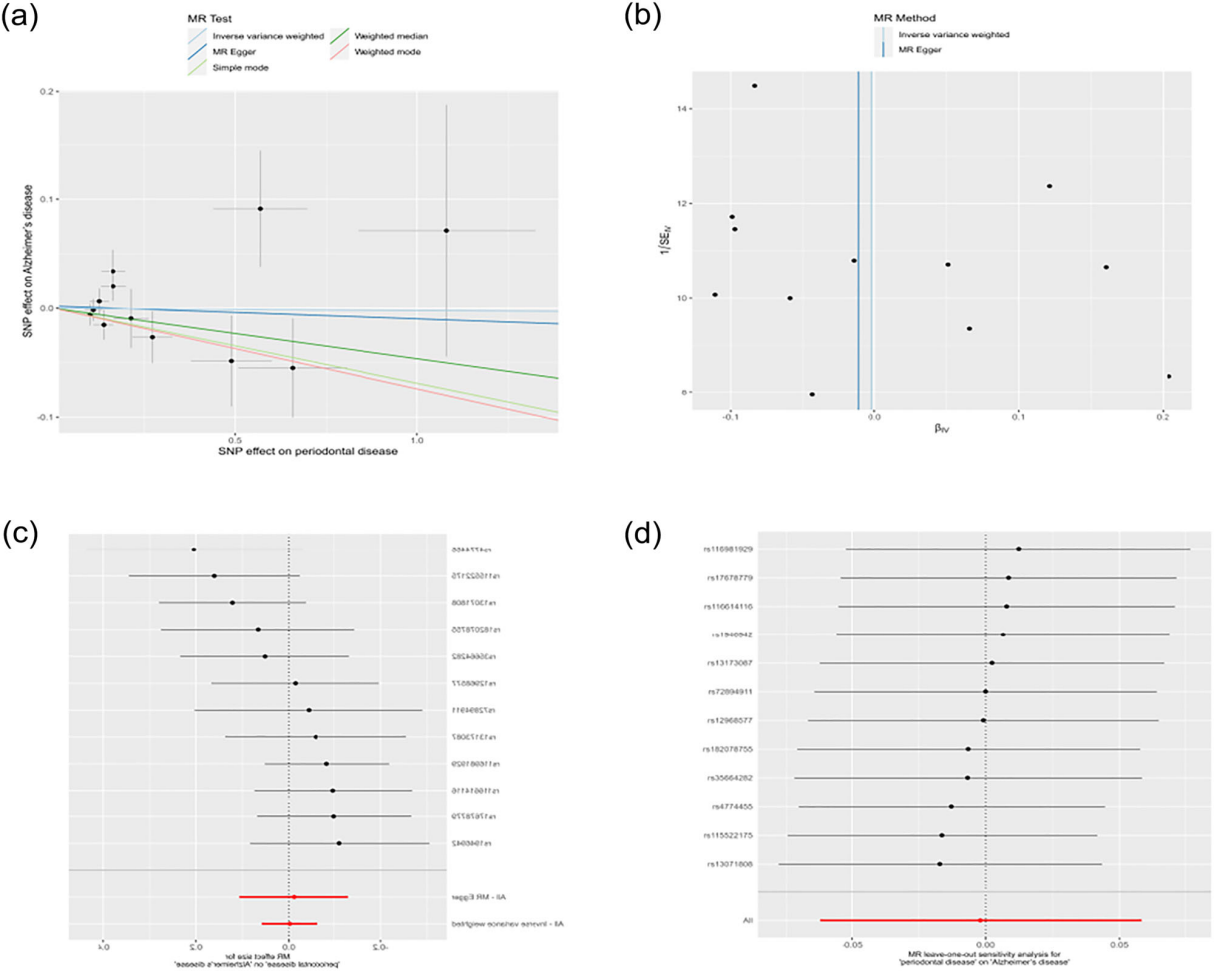
Mendelian randomization estimates from periodontal disease for AD (PGC). (a) Scatter plot showing the causality of periodontal disease on AD. (b) Funnel plots. (c) Forest plots of the IVW estimates. (d) Leave‐one‐out plots.

Consistent with previous methodologies, we employed IVW, MR‐Egger regression, and weighted median approaches to evaluate the causal relationship between periodontal disease and AD. The IVW analysis revealed no evidence of a causal link between periodontal disease and the risk of AD. Similarly, MR‐Egger regression and weighted median methods yielded concordant outcomes. Sensitivity analyses were conducted to ensure the robustness and integrity of the study. The Cochran *Q* test detected no indicators of heterogeneity (*Q* value = 14.55, *p*‐value = .204). Moreover, the horizontal pleiotropy test demonstrated a nonsignificant intercept (intercept = 0.002, *p* = .853), suggesting the absence of horizontal pleiotropy. A comprehensive summary of the horizontal pleiotropy test and the heterogeneity test can be found in Table 2. Outlier screening yielded no notable observations, hence the MR‐PRESSO method and Radial MR method were not applied. Leave‐one‐out analysis was also employed to assess the impact of each SNP on the overall causality estimation. Figure 3 illustrates the leave‐one‐out plot alongside additional findings.

**TABLE 2:** Sensitivity analyses of periodontal diseases IVs for AD (PGC).

Pleiotropy test			Heterogeneity test					
MR‐Egger			IVW			MR‐Egger		
Intercept	SE	*p*	*Q*	*Q*_df	*Q*_pval	*Q*	*Q*_df	*Q*_pval
0.002	0.0106	.853	14.549	11	0.204	14.497	10	0.152

MR, Mendelian randomization; IVW, inverse variance weighted; SE, standard error.

## 4 DISCUSSION

In this study, we employed a two‐sample Mendelian randomization (MR) approach to analyze the genetic causal relationship between periodontal disease and AD. The MR method enables causal analysis at the genetic level, providing direct insights into the genetic causal association between traits and diseases while circumventing issues encountered in traditional observational studies. Our findings indicated the absence of a genetic causal relationship between periodontal disease and AD. Although numerous studies have suggested a potential association between the pathogenesis of AD and periodontal disease, we did not find evidence supporting a causal link between these two conditions.

According to database‐based and large‐scale survey studies (Chen et al., 2017; Yu & Kuo, 2008), an association between periodontitis and cognitive decline has been identified. AD patients, due to their impaired ability to maintain oral hygiene, are more prone to developing periodontal disease (Laugisch et al., 2018). Autopsy studies have revealed the presence of *P*. *gingivalis* lipopolysaccharide in the gingiva of AD patients, but not in control subjects (Poole et al., 2013). Additionally, Dominy et al. (2019) demonstrated the presence of *P*. *gingivalis* DNA in the brain tissue of AD patients. Another study conducted by Riviere et al. (2002) observed higher levels of *Treponema* species in AD patients. These findings suggest that periodontal pathogens may enter the brain tissue from the oral cavity, triggering inflammation and ultimately leading to brain tissue damage (Mao et al., 2022). However, it is important to note that the detection of bacteria in the brain tissue, performed years after the patient's death, may result in bacterial contamination, and such contamination typically does not occur in the brain tissue of living AD patients (Emery et al., 2017).

Although epidemiological studies have confirmed an association between Alzheimer's disease (AD) and periodontal disease, the underlying mechanism remains unclear. Current research suggests three potential biological mechanisms linking periodontal disease and AD. First, periodontal disease‐induced systemic inflammation may result in the production of peripheral proinflammatory cytokines, such as C‐reactive protein, IL‐1, IL‐6, and tumor necrosis factor‐alpha. These cytokines can enter the brain through neural, humoral, and cellular pathways (Clive et al., 2017; Engelhart et al., 2004; Holmes et al., 2003; Schmidt et al., 2002). Second, periodontal bacteria or their molecules may directly enter the brain through the bloodstream or cranial nerves. Third, the pia mater could serve as a communication site between periodontal bacteria and microglia in the brain (Hashioka et al., 2019).Furthermore, studies examining periodontal pathogens have found associations between *P*. *gingivalis* levels and lower MMSE scores, as well as relationships between *Borrelia dentata* levels and concentrations of immune biomarkers (Leblhuber et al., 2020). These findings suggest that different microbial populations may have synergistic effects, altering the host immune response. However, research conducted by Lugisch et al. showed no significant differences in serum bacterial antibody levels between AD patients and those with other forms of dementia. Nevertheless, both groups exhibited higher levels of anti‐pathogen antibodies in their cerebrospinal fluid. This suggests that periodontal pathogens may trigger an immune response in the brain (Laugisch et al., 2018).It is important to note that the study by Lugisch et al. differs from the previously mentioned research by Dominy et al. Lugisch et al. did not detect the presence of bacteria in cerebrospinal fluid or serum samples but only identified specific antibodies. This indicates that only certain bacterial components may enter the brain or that the bacteria are exclusively present in brain tissue. In contrast, Dominy et al. (2019) reported the presence of *P*. *gingivalis* DNA in the brain and cerebrospinal fluid of AD patients. Additionally, studies have suggested that early‐onset AD and late‐onset AD may exhibit different immune responses to pathogens (Small et al., 1994), further adding to the complexity of understanding the relationship between periodontal disease and AD. These findings provide a potential link between periodontal disease and AD; however, further research is necessary to unravel the intricate mechanisms involved.

Current research primarily focuses on the epidemiological correlation between periodontal disease and Alzheimer's disease (AD), while studies specifically investigating the association between a particular pathogen and AD are limited. Currently, there is no study or experiment providing evidence that systemic infection caused by oral pathogenic bacteria precedes the pathological changes observed in AD. Additionally, the temporal relationship between the onset of periodontal disease and the pathological changes associated with AD remains unclear (Zong et al, 2018). Although the existing findings in this field of research suggest a potential link between periodontal disease and AD, further investigations are required to elucidate the underlying mechanisms and temporal sequence involved.

Furthermore, it is important to consider the potential contribution of genetic factors in the association between periodontitis and AD. Alzheimer's disease (AD) is a complex and multifactorial disorder with a strong genetic component. Numerous studies have identified several genetic variants that are associated with an increased risk of developing AD. These genetic variants include those involved in the production of beta‐amyloid, a protein that accumulates in the brain of individuals with AD, as well as genes related to inflammation, immune response, and lipid metabolism.

Interestingly, recent research has also suggested a potential link between these genetic risk factors for AD and the development and progression of periodontitis. For instance, certain genetic variants implicated in AD have been found to be associated with an increased susceptibility to periodontitis. Additionally, studies have shown that individuals with AD may have a higher prevalence and severity of periodontitis compared to those without the disease.

The potential genetic overlap between periodontitis and AD raises intriguing questions about the shared underlying biological mechanisms. It is possible that common pathways, such as inflammation and immune dysregulation, contribute to the pathogenesis of both diseases. Furthermore, chronic periodontal inflammation may have systemic effects on the brain, potentially influencing the progression of neurodegenerative conditions like AD.

However, despite these observations, it is important to note that the precise nature of the genetic relationship between periodontitis and AD remains poorly understood. The complex interplay between genetic and environmental factors in the development and progression of both diseases adds another layer of complexity to the puzzle. Future studies using large‐scale genetic analyses, as well as functional experiments and animal models, are needed to unravel the exact genetic mechanisms at play and to determine the causal relationship between periodontitis and AD.

In conclusion, while there is emerging evidence suggesting a potential genetic link between periodontitis and AD, further research is required to fully elucidate the underlying genetic factors connecting these two conditions. Understanding the genetic basis of the association between periodontitis and AD could have important implications for both disease prevention and treatment strategies.

Of course, the study has its limitations. First, our study only included populations from the European region, so caution should be exercised when extrapolating our findings to populations from other regions of the world. Second, gender differences were not taken into account in our study. Therefore, careful consideration should be given to potential variations in our conclusions when applied separately to male or female populations. Additionally, our study was conducted solely at the genetic level, and the possibility of other factors contributing to the association between the two diseases cannot be completely ruled out.

## 5 CONCLUSIONS

Our findings indicate that there is no causal relationship between periodontal disease and AD at the genetic level. However, this does not completely exclude the possibility of their association at nongenetic levels. Further extensive and in‐depth studies are required to investigate these potential connections.

## AUTHOR CONTRIBUTIONS

**Conglei Hu**: Conceptualization; methodology; software; data curation; formal analysis; writing—original draft. **Liping Huang**: Conceptualization; methodology; software; data curation; formal analysis; writing—original draft. **Hui Li**: Conceptualization; methodology; software; data curation; formal analysis; funding acquisition. **Rui Wang**: Conceptualization; formal analysis; methodology; software; data curation; writing—original draft. **Zeyu Wang**: Conceptualization; methodology; software; data curation; formal analysis; writing—original draft. **Rui Ma**: Conceptualization; methodology; software; data curation; formal analysis; writing—original draft. **Bei Chang**: Conceptualization; methodology; software; data curation; formal analysis; writing—original draft. **Shiting Li**: Conceptualization; methodology; software; data curation; formal analysis; writing—original draft. **Hongcai Li**: Conceptualization; methodology; software; data curation; formal analysis; writing—original draft. **Guangwen Li**: Supervision; funding acquisition; writing—review and editing.

## CONFLICT OF INTEREST STATEMENT

The authors declare no conflict of interest.

### PEER REVIEW

The peer review history for this article is available at https://publons.com/publon/10.1002/brb3.3486.

## Supporting information

Supporting Information

## Data Availability

Publicly available datasets were analyzed in this study. These data can be found here: IEU OpenGWAS Project (https://gwas.mrcieu.ac.uk, accessed on August 30, 2023) and Psychiatric Genomics Consortium (https://pgc.unc.edu, accessed on August 30, 2023).
