# Supplementary material for: Periodontal disease and risk of Alzheimer's disease: A two‐sample Mendelian randomization
Source: Brain Behav. 2024 Apr 22;14(4):e3486. doi: 10.1002/brb3.3486 (PMC11034860; doi:10.1002/brb3.3486)
Supplement: Supplementary file 1 — Supporting Information [file BRB3-14-e3486-s001.docx]

**Contribution of Authors**

This article was written by Conglei Hu, who independently completed the study design and data analysis. In addition, Hui Li and Liping Huang led the writing of the article and proofread all drafts. In terms of revision of the paper, Guangwen Li objectively revised the article. All authors contributed to further revisions of this article. Given the heterogeneity of knowledge in the study, Rui Wang, Bei Chang, Shiting Li and Hongcai Li were involved in the study design, key decisions, data collection, and preliminary report. In terms of data analysis, Rui Ma and Zeyu Wang is responsible for testing and analysis, and is centrally responsible for statistical data processing to ensure data accuracy. The data was confirmed by Conglei Hu. Finally, all the authors contributed to the writing and revision of the article. All study members received consistent study participant information and results reports based on documentation.
